# Factors associated with pneumococcal vaccination uptake in over 50s in Ireland: a cross-sectional study using results from the Irish Longitudinal Study on Ageing (TILDA)

**DOI:** 10.1136/bmjph-2025-003996

**Published:** 2026-03-31

**Authors:** Kevin Organ, Siobhan Scarlett, Cathal McCrory, Sinead Mc Loughlin, Ann Hever, Rose Anne Kenny

**Affiliations:** 1 The Irish Longitudinal Study on Ageing (TILDA), Trinity College Dublin, Dublin, Ireland; 2 St James's Hospital Mercer’s Institute for Successful Ageing, County Dublin, Ireland

**Keywords:** Vaccination, Public Health, Preventive Medicine

## Abstract

**Introduction:**

Pneumococcal vaccination is a key global health strategy to reduce the morbidity and mortality associated with pneumococcal disease.

**Methods:**

This study examined the factors associated with pneumococcal vaccination uptake in community-dwelling older Irish adults using data from Wave 5 of The Irish Longitudinal Study on Ageing. For unvaccinated individuals, we also examined the factors associated with the likelihood of health professionals having discussed pneumococcal vaccination with them. Both relationships were modelled using Poisson regression.

**Results:**

Higher pneumococcal vaccination uptake was associated with having a history of previous influenza vaccination (incidence risk ratio, IRR=9.08; 6.69 to 12.32, p<0.001), being part of an at-risk medical group (IRR=1.41; 95% CI 1.26 to 1.58, p<0.001) and having reduced barriers to healthcare access with entitlement to a medical card or general practitioner visit card (IRR=1.65; 95% CI 1.08 to 2.51, p<0.05). For those who are unvaccinated, a higher likelihood of receiving medical advice regarding pneumococcal vaccination was seen in females (IRR=1.69; 95% CI 1.34 to 2.12, p<0.001), those aged 65–74 years (IRR=1.70; 95% CI 1.27 to 2.27, p<0.001) and those with an at-risk medical condition (IRR=1.58; 95% CI 1.25 to 2.00, p<0.01). Conversely, frail individuals compared with non-frail individuals were less likely to have received a pneumococcal vaccination recommendation (IRR=0.53; 95% CI 0.30 to 0.94, p<0.05).

**Conclusions:**

Pneumococcal vaccination uptake in eligible older Irish adults remains low, highlighting a significant unmet need. Targeted interventions to increase recommendations of the vaccination by healthcare professionals may help with reducing pneumococcal disease burden.

**WHAT IS ALREADY KNOWN ON THIS TOPIC**

- Vaccination of older adults can reduce the disease burden associated with pneumococcal disease, but both uptake rates and eligibility criteria differ between countries.

**WHAT THIS STUDY ADDS**

- We examined the interplay between Ireland’s mixed public–private healthcare provision model and the barriers this has on the implementation of Ireland’s national immunisation strategy.
- We were able to combine the health and economic data for a nationally representative sample of older Irish adults to look at drivers of vaccination uptake within this cohort.

**HOW THIS STUDY MIGHT AFFECT RESEARCH, PRACTICE OR POLICY**

- Ireland needs a vaccination strategy that eliminates the financial barriers to vaccination in order to achieve adequate coverage and reduce the associated disease burden.
- Healthcare providers need to increase their recommendations for pneumococcal vaccinations to older adults, particularly those with frailty who are currently being missed.

## Introduction

 Pneumococcal disease is composed of symptomatic infections caused by the bacterium *Streptococcus pneumoniae* and is associated with several infections such as sinusitis, community-acquired pneumonia and more severe invasive pneumococcal diseases such as meningitis and sepsis.^1^

Pneumococcal disease presents a significant global health burden, with a high degree of mortality and morbidity associated with pneumococcal disease in very young children, older adults and younger adults with certain chronic health conditions.^1^ Pneumococcal disease also presents great challenges for older adults with frailty, as frail individuals hospitalised with community-acquired pneumonia needed a longer duration of antibiotic treatment, had longer inpatient stays and had a higher overall risk of mortality compared with robust individuals.^2^ Data using the Global Burden of Diseases studies estimated that pneumococcal pneumonia, or pneumonia caused by *S. pneumoniae,* killed 218 540 people aged ≥70 years globally in 2021.^3^ Similarly, the Central Statistics Office Vital Statistics Annual Report 2019 found that 1017 deaths in those aged ≥65 years were attributed to influenza and pneumonia in Ireland.^4^

Pneumococcal disease also presents a high economic burden across various direct and indirect costs such as inpatient care, outpatient care and lost workdays. The financial burden in Europe from pneumonia costs approximately €10.1 billion annually, with €3.6 billion of this being associated with lost workdays.^5^ This is a growing concern as the proportion of older adults increases globally. It is projected that the percentage of the population aged over 65 will reach 16% in 2050 compared with a rate of 9.3% in 2020.^6^ This demographic shift could result in a much higher economic burden.

### Vaccination as a tool

Vaccination is one of the primary strategies to mitigate the global burden of pneumococcal disease.

The WHO’s Immunisation Agenda 2030 framework underscores this importance, emphasising the need to enhance vaccination awareness and uptake throughout the life course.^7^ Indeed, many countries currently offer pneumococcal vaccination to infants, older adults or those with various chronic health conditions or immunocompromising conditions.^8^ The two primary vaccines for pneumococcal disease are the pneumococcal conjugate vaccine (PCV) and the pneumococcal polysaccharide vaccine (PPV).^1^

In Ireland, the National Immunisation Advisory Committee (NIAC) provides guidelines for pneumococcal vaccination. Under these guidelines, the PCV13 is currently administered to infants as part of the primary childhood immunisation schedule.^9 10^ The PPV23 vaccine is also recommended for all adults aged over 65 years or individuals under 65 with a medical condition associated with an increased risk of invasive pneumococcal disease, such as asplenia, chronic heart, lung or liver disease, etc.^9^ If a person aged under 65 years receives a vaccination, a second dose is recommended after age 65, provided 5 years have passed since receiving their first dose.^9^

Data from the Health Service Executive (HSE) Health Protection Surveillance Centre Annual Epidemiological report on invasive *S. pneumoniae* for 2018 indicates a significant reduction in disease burden following the introduction of the PCV.^11^ The report further identified that several predominant pneumococcal serotypes in circulation were not covered by the PCV13 vaccine but were included within the PPV23 vaccine. Due to this, the PPV23 vaccination remains recommended for older and at-risk groups, despite its lower efficacy compared with the PCV13 in reducing overall pneumococcal disease burden.^11^

### International uptake and drivers

Pneumococcal vaccination is widely available, yet rates of vaccination uptake in older adults vary substantially between countries. Uptake can be as low as 20.1% in Israel and as high as 71.0% in the USA for those aged 65 and older.^12^ This variation may stem from differing eligibility in immunisation guidelines. A recent review highlighted significant heterogeneity in both age-based and risk-based vaccination guidelines across Europe, with age recommendations ranging from 50 years in Austria and Poland to 65 years in the UK and Ireland.^8^ The risk-based guidelines were found to be similarly inconsistent, with asplenia being the only condition universally included in the vaccination guidelines of the European countries studied.^8^

### Ireland’s mixed healthcare model

One factor influencing healthcare utilisation in Ireland is the unique mixed healthcare provision model, which is outlined by the Health Acts 1947 to 2022.^13^ This is a largely means-tested system that divides the population into two groups; those entitled to a medical card or a general practitioner (GP) visit card and those without these entitlements. Medical cards give service users free access to a range of primary care services, as well as reduced prescription costs, while GP visit cardholders receive free service at point of access but still pay full prescription and other associated healthcare costs. This means test is assessed using a qualifying threshold of a person’s net income and assets with allowable deductions for essential expenses such as rent or mortgage payments, childcare costs or reasonable expenses for travelling to work, etc.^14^ While medical cards are a means-tested entitlement, some groups qualify for a medical card without a means test.^15 16^ There are also some groups with an automatic entitlement to GP visit cards, such as children under 8 years, those aged over 70 and those receiving carers allowance payments.^16^ Those without either card must cover all associated healthcare expenses, such as GP fees.^17^ Mc Hugh *et al*^18^ assessed how this mixed healthcare model impacts the rates of influenza vaccination, finding that adults over 50 with a medical card were nearly twice as likely to receive an influenza vaccination than those without.

Irish individuals can also receive private health insurance coverage, which generally covers hospital treatment, some outpatient care and partial refunds for specialist visits, scans and preventive services like vaccinations.^19^ The coverage for vaccinations and GP consultations can vary depending on individual health insurance plans, with one, for example, covering 50% of vaccination costs up to €50 per year plus 75% of GP visit costs^20^ and another covering influenza vaccinations up to €30 per year plus 75% of GP visit costs up to €60 per visit.^21^

Research on pneumococcal vaccination uptake for specific medical conditions in Ireland is relatively limited.^22^ Giese *et al*^23^ conducted telephone interviews in 2013, finding that overall pneumococcal vaccine uptake was low, with only 28% of Irish adults between the ages of 18–64 with an at-risk medical condition being vaccinated and 36% of those aged 65+ in this group being vaccinated. The study focused on younger but medically vulnerable groups as this data often goes unreported; however, education and private insurance were not accounted for, and the data predated pharmacy administration of the vaccination.

This study aims to use the rich data captured by The Irish Longitudinal Study on Ageing (TILDA) to identify the prevalence and characteristics of older adults living in Ireland who have received the pneumococcal vaccination based on both age-based and risk-based eligibility. A number of potential factors associated with uptake of the vaccine will also be explored, including healthcare entitlements, health conditions and sociodemographic characteristics. The findings aim to identify any unmet needs and to inform targeted vaccination strategies that may increase the uptake among older Irish adults.

## Methods

### Study design and population

Data were from TILDA. TILDA is a prospective nationally representative longitudinal study of community-dwelling adults aged 50 years and older, and their spouses of any age, in the Republic of Ireland.^24^,^26^ The study captures detailed information on the health, social and economic circumstances of each participant. The first wave of data collection took place between October 2009 and February 2011 with follow-up data collection periods approximately every 2 years after. The sampling fraction is approximately 1/156 of all community-dwelling adults aged 50 years and over resident in the Republic of Ireland.^24 25 27^ The sample was generated using a two-stage clustered sampling process and the Irish Geodirectory as the sampling frame, which comprises all addresses in Ireland. The primary sampling units were 640 geographic regions selected by random selection, stratified on proportion of head of households in the professional class, proportion of the population aged 65 and older and geographical location. The second stage involved the selection of a random sample of 40 addresses from within each primary sampling unit, resulting in an initial sample of 25 600 addresses, which were assessed for eligible participants aged 50 and older. A response rate of 62.0% (n=8504, 8175 aged 50+) was achieved at the household level, which was defined as the proportion of sampled households, including an eligible participant from whom an interview was successfully obtained.^24 25 27^ Participation in TILDA is voluntary and a full description of the study has been described elsewhere.^24^

### Sample

**Figure 1:**
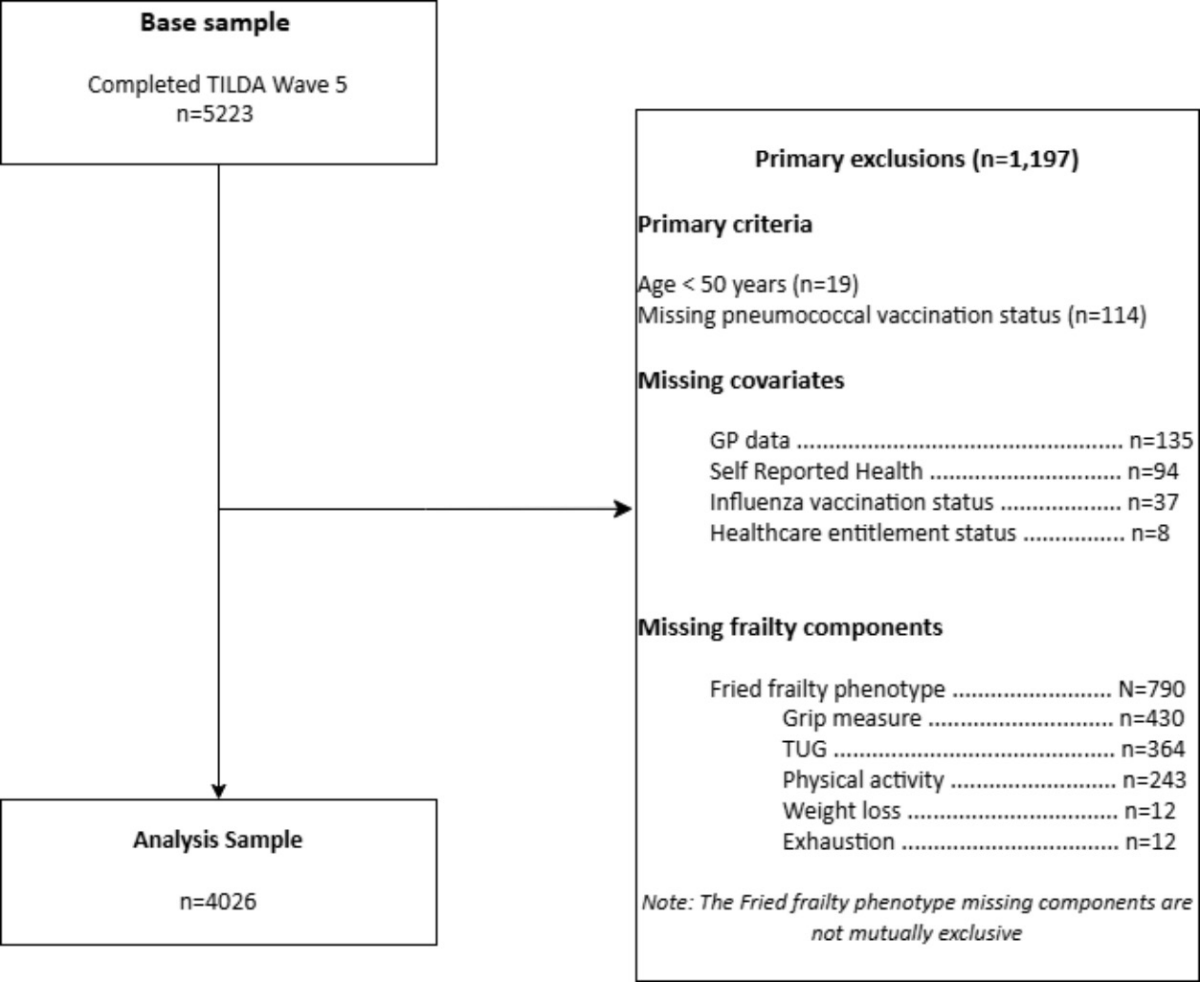
Flow diagram depicting the derivation of the analytical sample. GP, general practitioner; TILDA, The Irish Longitudinal Study on Ageing; TUG, Timed Up and Go test.

This analysis used data from wave 5 of TILDA (January–December 2018). 5223 TILDA participants took part in this wave.^28^ The final analysis sample (n=4026) excluded anyone under 50 years of age or who had missing data for any analysis measures (figure 1). The majority of these drops (n=790) were due to respondents missing one or more components of the Fried frailty phenotype during this wave of data collection. As the drops caused by the inclusion of the Fried frailty phenotype represent a 15% reduction in sample size, a sensitivity analysis was performed with this variable excluded and the larger sample retained (online supplemental table S1; online supplemental table S2). As the other associations remained largely stable, we retained the Fried frailty phenotype in our analysis.

### Measures

#### Pneumococcal vaccination status

Participants were asked to self-report whether they have ever received a pneumococcal vaccination in the past (No/Yes).

#### Vaccination recommendation by healthcare practitioners

Participants who did not receive the vaccine were asked which, if any, healthcare providers discussed pneumococcal vaccination with them. A dichotomous variable was generated to capture whether vaccination was discussed by any of the providers (not discussed/discussed).

#### At-risk medical conditions

Participants were asked at each wave to self-report if they had received a doctor diagnosis of a range of health conditions. These conditions are then confirmed again at each subsequent wave to flag any misdiagnoses or recording errors, which undergo systematic removal. The Irish pneumococcal vaccination recommendations disseminated by the HSE^29^ and Royal College of Physicians of Ireland (RCPI)^9^ were used to identify which medical conditions would class somebody as having an at-risk medical condition. Confirming the presence of one or more of the at-risk conditions which were available (chronic obstructive pulmonary disease, lung disease, diabetes, cancer, liver disease, kidney disease, immune deficiency, neurological condition, heart attack and heart failure) marked the participant as being in an at-risk medical group (not at risk/at risk). This group would be eligible for free vaccination. Age-based eligibility will be examined independently for our analysis.

#### Previous influenza vaccination uptake

Participants were asked if they had ever received an influenza vaccination (No/Yes).

#### Healthcare entitlement

Participants were asked about healthcare entitlements and were categorised as having no health coverage; private health insurance only; a medical card or GP visit card only; or dual coverage (both private health insurance and a medical card or GP visit card).

#### Self-rated physical health

Participants were asked to self-report their physical health status (excellent/very good/good/fair/poor).

#### Distance to nearest GP

GP distance was calculated and derived as quantiles using road network distance measures from each respondent’s home co-ordinates to their nearest GP practice.^30^ The lowest quantile represents the closest proximity to GP access.

#### Fried frailty phenotype

Frailty was classified using the Fried frailty phenotype.^31^ Individuals were classed as either non-frail (0 components), prefrail (1–2 components) or frail (3–5 components).

### Covariates

Covariates known to impact the likelihood of receiving a vaccination were controlled for: age (50–64 years; 65–74 years; 75+years), sex (male/female), marital status (married, never married, separated/divorced, widowed) and education (primary/none, secondary, third/higher).

### Statistical analysis

All analyses were performed using Stata V.18.^32^ Associations between pneumococcal vaccination uptake and potential driving factors were modelled to ascertain which factors affect the likelihood of having received a pneumococcal vaccination. We then modelled the factors associated with whether unvaccinated participants had the pneumococcal vaccination discussed with them by a healthcare provider.

For both models, a modified Poisson regression with robust standard errors was chosen as outlined by Zou.^33^ Although the Poisson model assumes equidispersion, when applied to binary outcomes, the robust variance estimator corrects for variance misspecification, allowing consistent estimation of risk ratios. This approach is widely recommended when outcomes are common, as ORs from logistic regression may overestimate the relative risk.^34^ This model approach was commonly used in other vaccination research.^35 36^

A number of diagnostics were performed to ensure that this was the most appropriate model for our analysis. During our preliminary research, a likelihood ratio test was conducted on our unweighted models comparing the Poisson model to a negative binomial model. The tests indicated no evidence of overdispersion (X^2^=0.00, p=1.000) and thus a Poisson model was chosen.

We assessed multicollinearity using variance inflation factors (VIF) on the full sample (N=4016). Factor variable notation in Stata was used for categorical variables. VIF values ranged from 1.07 to 4.14 with a mean VIF value of 1.92.

As this analysis utilises survey weights, both models were then assessed using the manual link test. Our model for pneumococcal vaccination uptake showed a significant result (p=0.003), so a further sensitivity analysis was performed using a negative binomial regression and a logistic regression (online supplemental table S3). The direction and statistical significance of associations were unchanged, supporting the robustness of our findings. Given our interest in directly estimating risk ratios for a common outcome, we retained the modified Poisson specification.

Results are presented as incidence risk ratios (IRR) with 95% CIs. IRRs indicate the relative risk of the outcome associated with each predictor, where a value below 1 suggests a negative association and a value above 1 suggests a positive association.

Statistical significance was set at p<0.05.

### Strengthening the Reporting of Observational Studies in Epidemiology reporting

We used the STROBE (Strengthening the Reporting of Observational Studies in Epidemiology) reporting guideline^30^ to draft this manuscript, and the STROBE reporting checklist when editing, which has been included as a supplementary file.

## Results

Descriptive characteristics by pneumococcal vaccination status are presented in table 1.

**Table 1:** Demographic characteristics by pneumococcal vaccination status

	Vaccinated n=92	Not vaccinated n=3103	Total n=4026
	%	%	%
Overall	24	76	100
Sex			
Male	42.3	51.2	49.1
Female	57.7	48.8	50.9
Age group			
50–64 years	13.0	39.1	32.9
65–74 years	42.1	39.2	39.9
75+ years	44.9	21.7	27.2
Marital status			
Married	57.2	69.2	66.3
Never married	10.2	10.1	10.1
Separated/divorced	6.0	8.8	8.2
Widowed	26.6	11.9	15.4
Level of education			
Primary/none	33.2	24.2	26.3
Secondary	44.8	45.4	45.3
Third/higher	22.0	30.4	28.4
Self-rated health			
Excellent	7.8	13.2	11.9
Very good	29.2	37.1	35.2
Good	42.4	34.4	36.3
Fair	16.3	13.1	13.9
Poor	4.3	2.2	2.7
At-risk groups			
Not at risk	56.6	76.8	72.0
At risk	43.4	23.2	28.0
Influenza vaccination			
No	5.8	56.1	44
Yes	94.2	43.9	56
Healthcare entitlement			
No cover	2.4	9.5	7.8
Insurance only	11.2	35	29.2
Medical card/GP visit card only	41.9	32.9	35.1
Dual coverage	44.5	22.6	27.9
Eligibility*			
Eligible	95.2	68.7	75.0
Ineligible	4.8	31.3	25.0
GP distance			
First quantile (closest)	25.9	20.3	21.6
Second quantile	23.9	21.6	22.2
Third quantile	18.9	21.8	21.1
Fourth quantile	17.1	18.2	17.9
Fifth quantile (furthest)	14.2	18.1	17.2
Frailty			
Non-frail	38.2	56.2	51.9
Prefrail	47.6	36.5	39.2
Frail	14.2	7.3	8.9

* Eligibility is inclusive of those aged over 65 years and the at-risk group.

GP, general practitioner.

The majority of the sample were aged 65 years and older (67.1%) with a mean age of 69.9 years, and 50.9% were female.

Just 7.8% of the sample reported no healthcare entitlement, with the remainder having access to private health insurance only (29.2%), a medical card/GP visit card (35.1%) or both (27.9%). Only 28.0% were categorised as ‘at risk’; however, 75.0% were eligible for the pneumococcal vaccination based on age thresholds and the at-risk criteria. Over half the sample (56%) reported receiving the influenza vaccination previously.

Prevalence of pneumococcal vaccination in the sample overall was low (24.0%). Of those who are vaccinated, 63.1% were vaccinated by their GP, 34.0% were vaccinated by a nurse in their GP practice and 0.9% were vaccinated by a pharmacist (data not shown).

An age trend was apparent, with the highest prevalence of vaccination among those aged 75 years and older (44.9%). For the combined total of all respondents aged 65 and older, the overall uptake was 31.1%. Of all individuals aged between 50 and 64 years, 13.0% received a pneumococcal vaccination, yet 25.3% of this age cohort were eligible under the risk-based guidelines. Of this eligible group, the vaccination uptake was 23.9%.

Frail individuals made up 14.2% of the vaccinated group compared with 7.3% of the unvaccinated group. Those classed as at-risk made up 43.4% of the vaccinated group compared with 23.2% of the unvaccinated group.

Previous vaccinations and healthcare entitlements appeared to play a role in likelihood of vaccination, with 94.2% of those in the vaccinated group having received a previous influenza vaccination. Those with no healthcare entitlement made up 2.4% of the vaccinated group compared with 9.5% of the unvaccinated group. In contrast, those with dual healthcare coverage made up 44.5% of the vaccinated group compared with 22.6% of the unvaccinated group. Notably, those with medical insurance coverage only made up 35% of the unvaccinated group compared with only 11.2% of the vaccinated group. Among respondents with private health insurance, 20.8% reported receiving only partial reimbursement for GP consultation costs, whereas 71.1% reported receiving no reimbursement for these fees (data not shown).

Trends of uptake were also apparent relative to proximity to GP. Those in the closest proximity to a GP made up 25.9% of the vaccinated group compared with 20.3% of the unvaccinated group, while just 14.2% of those furthest from a GP were in the vaccinated group compared with 18.1% in the unvaccinated group.

### Factors associated with pneumococcal vaccination uptake

The weighted Poisson model demonstrated significant explanatory power (F(23, 574)=26.62, p<0.001).

**Figure 2:**
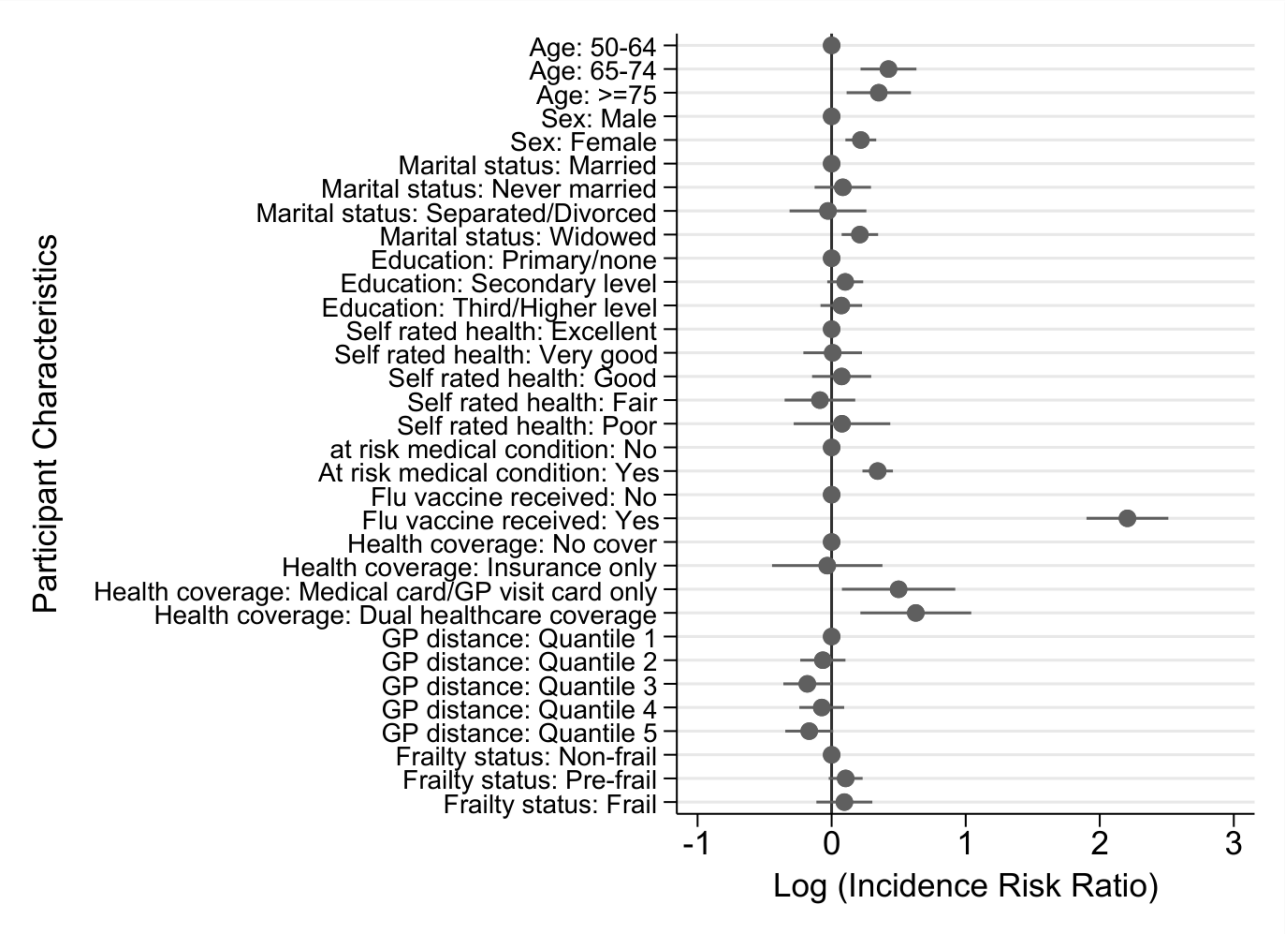
Poisson regression modelling associations between participant characteristics and pneumococcal vaccination uptake. GP, general practitioner.

Having received an influenza vaccination in the past was significantly associated with a higher likelihood of having also received a pneumococcal vaccination (IRR=9.08; 95% CI 6.69 to 12.32, p<0.001) (figure 2).

Those with an at-risk medical condition were also more likely to be vaccinated (IRR=1.41; 95% CI 1.26 to 1.58, p<0.001) compared with those without one of these health conditions.

There was a significant positive association among those with a medical card/GP visit card (IRR=1.65; 95% CI 1.08 to 2.51, p<0.05) or those with dual healthcare coverage (IRR=1.87; 95% CI 1.24 to 2.83, p=0.01) having a higher vaccination likelihood compared with those without any healthcare entitlements.

### Factors associated with a healthcare professional’s likelihood to discuss pneumococcal vaccination

The weighted Poisson model demonstrated significant explanatory power (F(23, 566)=3.77, p<0.001).

Of those who reported either being unvaccinated or unsure of their pneumococcal vaccination status (n=3087), 89.9% have not had any healthcare practitioners discuss the option of vaccination with them.

**Figure 3:**
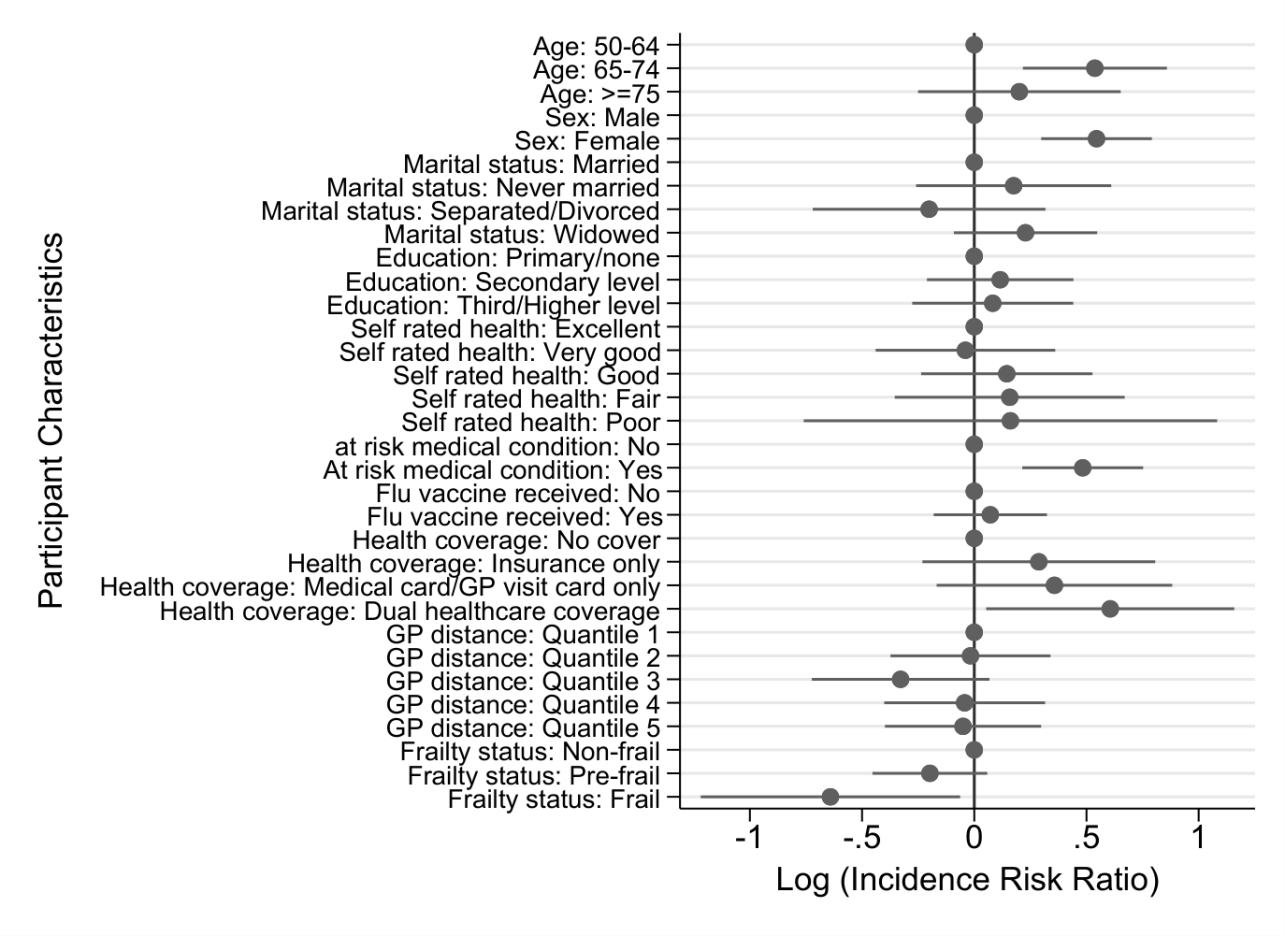
Poisson regression modelling associations between participant characteristics and whether a healthcare professional spoke to them about pneumococcal vaccination. GP, general practitioner.

Females were more likely to have vaccination discussed with them by a healthcare provider compared with males (IRR=1.72; 95% CI 1.35 to 2.21, p<0.001), as were those aged 65–74 years compared with those aged 50–64 years (IRR=1.71; 95% CI 1.24 to 2.36, p<0.01) (figure 3).

Those with an at-risk medical condition were significantly more likely to have discussed pneumococcal vaccination with a healthcare provider (IRR=1.62; 95% CI 1.24 to 2.12, p<0.001) compared with those not at risk.

It was also found that unvaccinated frail individuals were less likely to have been recommended a pneumococcal vaccination by their medical providers (IRR=0.53; 95% CI 0.30 to 0.94, p<0.05) compared with robust individuals.

When running our sensitivity analysis (online supplemental table S2) comparing the primary model to a model with the Fried phenotype excluded, we can see that the association between having dual medical coverage does not reach statistical significance (IRR=1.63; 95% CI 0.99 to 2.67) when compared with the analysis retaining the Fried phenotype (IRR=1.83; 95% CI 1.05 to 3.19). However, the broad overlap between the two intervals and the relative stability of the point estimates suggests that the underlying association remains broadly consistent across both models.

## Discussion

This research explored factors influencing the uptake of pneumococcal vaccination among older adults in Ireland. To our knowledge, we were the first study to analyse pneumococcal vaccination uptake in older Irish adults while accounting for both medical and age-based risk guidelines as well as incorporating a measure of phenotypic frailty to assess vaccination uptake and vaccine recommendation in those most at risk of morbidity and mortality.

Despite a significant proportion of adults aged over 50 being eligible for pneumococcal vaccination, the uptake remains very low with eligible older adults comprising 68.7% of the unvaccinated group. When comparing our data to Giese *et al*^23^ we see only a modest increase in uptake of 7.9% for the younger medical risk groups and a conversely decreasing trend of almost 5% for the over 65 groups. This discrepancy regarding the rates of uptake may be attributed to differences in study design, with telephone interviews being more prone to social desirability bias.^37^ Our longitudinal cohort study may also be more well suited to capture individuals with lower levels of health engagement and thus give a more accurate representation of vaccine uptake in older Irish adults. This is because we repeatedly capture our participants’ healthcare entitlement status over time in combination with prospective data on healthcare utilisation patterns, such as GP visits across each wave. The lower rates among the younger risk groups may also be partially attributed to the fact that their risk cohort is for those aged 18–64, whereas ours was targeted to the 50–64 age group. Given the relative similarity in rates of uptake, this indicates that overall uptake among Irish adults remained relatively stable between the two study periods.

The strongest predictor of pneumococcal vaccination uptake was prior influenza vaccination, suggesting that targeting this group could likely boost pneumococcal vaccination rates. This finding supports similar previous research that noted a positive association between prior influenza vaccination uptake and individual likelihood to be vaccinated for pneumonia.^23 38 39^ As the influenza vaccine is routinely offered annually, this also presents a series of repeated opportunities for healthcare practitioners to discuss or offer pneumococcal vaccination. This would particularly be effective for individuals who are aged over 65, as this entire cohort is eligible. Indeed, influenza and pneumococcal vaccines can be given concurrently,^29^ offering opportunities for a co-vaccination approach as a simple intervention to increase vaccination rates among older groups. This type of recommendation has already been implemented by the NIAC in both their COVID-19^40^ and their influenza^41^ vaccination guidance.

This study has also identified one of the most significant predictors of vaccination uptake is a person’s individual healthcare entitlements under the Irish mixed healthcare model. Those receiving care for free at point of service were more likely to be vaccinated than those with no medical coverage or only a private health insurance plan. This matches previous findings for influenza vaccination^18^ and builds on the findings from Giese *et al*^23^ who found a similar association between pneumococcal vaccination uptake and free GP visits. With our study also having data on private health insurance information, we could see that the majority of those with private health insurance reported that they either receive no reimbursement or only a partial reimbursement for the costs of GP practice consultation fees. This further suggests that upfront cost is a key barrier driving the low levels of vaccination uptake in older adults, as on average, GP visit fees can be as much as €50–€75 per appointment.^42^ This is an important finding, as while somebody could be medically entitled to free vaccination under the Irish immunisation guidelines, they may still incur medical consultation costs, thus preventing them from availing themselves of this service. This may also present a dual disadvantage where the recommendation of the vaccine is even less likely to occur due to a lower frequency of GP visits by these individuals. The lower numbers of GP visits in these groups may cause cumulative reductions in the opportunity of these patients to receive appropriate vaccination recommendations and guidance.

There are a number of possible ways that this could be addressed, with one such strategy being to make pneumococcal vaccinations free at source. This type of scheme would cover all costs associated with the service, such as consultation fees and would thus eliminate this barrier to vaccination uptake. Such a model has been implemented as part of the Free Contraception Scheme which was launched in 2022 and reduced the cost barriers to several products such as prescription contraception and contraceptive implants.^43^

Another possible solution to tackle this issue could be to set up vaccination centres that would offer free pneumococcal vaccination to older adults. We have seen this utilised successfully in a large scale during the COVID-19 pandemic as part of the COVID-19 Vaccination Strategy^44^ and on a much smaller scale during the Mpox outbreak to target key at-risk groups during the spread of this infection.^45^ While both the COVID-19 and Mpox outbreaks were much more emergent and publicised public health crises, they have shown that alternative delivery models are feasible in the Irish context and could warrant consideration. The above options would likely be subject to a Health Technology Assessment by the Health Information and Quality Authority to assess a number of factors, such as the financial costs, clinical efficacy and organisational issues for such a rollout.^46^

A concerning finding from this study was the significant lack of discussion between healthcare practitioners and older adults regarding vaccination. While the responses overall were very low, with 89.9% not having had vaccine discussions with a primary care provider, this impact was most acutely seen for individuals who are classed as frail, who had a lower likelihood of receiving healthcare advice regarding the benefits of pneumococcal vaccination. As this group is the most at risk of negative outcomes from pneumococcal infection, such as increased hospital stays and a higher mortality risk,^2^ they should be a key target for vaccination. There are a number of reasons that may contribute to this low rate of recommendation. As our chosen frailty measure was the Fried frailty phenotype, which uses functional frailty measures such as exhaustion and slowness, this may have indicated a lack of knowledge around recognising frailty in the context of a standard consultation. If a frail individual attends their GP for an acute illness or infection, vaccination may not be discussed as it would not be feasible to administer them during this visit. This is reflected in the NIAC guidelines that advise deferral until recovery for febrile illness.^9^

This analysis also revealed a small yet significant effect of GP distance on a person’s likelihood to receive a pneumococcal vaccination. This could warrant further study in a longitudinal format to ascertain what effect it may have regarding uptake for several other adult vaccinations such as those for influenza and varicella-zoster, which are also recommended for older Irish adults.

As wave 5 was the first to include specific questions around pneumococcal vaccination, future research could examine trends in uptake using longitudinal data from subsequent waves of TILDA. Wave 6, conducted between September 2020 and December 2021, could provide insights into the impact of the COVID-19 pandemic on vaccination rates for both influenza and pneumonia using wave 5 data as a baseline comparator, and wave 7 can be used to analyse the post COVID-19 vaccination landscape.

This study had a number of strengths. The data was from a large, representative study of older adults in Ireland. This cross-sectional analysis was performed on a longitudinally validated cohort to assess the drivers and barriers to pneumococcal vaccination in older Irish adults. Our health data have been gathered across five waves, allowing us to retroactively audit our self-reported medical data for any recording errors or misdiagnoses. Ireland’s mixed healthcare model also gave an opportunity to examine the differences faced by those receiving free medical care at point of service vs those who pay privately either through health insurance or out-of-pocket payments.

Limitations were also present. Our analyses were cross-sectional, meaning trends in uptake over time were not analysed. The question around whether a healthcare professional had discussed the vaccination was only asked to those who had not received the vaccination, limiting our ability to assess the impact this had on those who did receive the vaccination. If this question regarding prior discussion of the vaccine had been posed to the entire cohort, we may have been able to assess the independent impact that vaccination advice has on pneumococcal vaccination uptake, as this was a significant predictor as analysed by other studies.^23 47 48^ This can be addressed in future research, as the survey methodology was amended for future waves to ask both the vaccinated and unvaccinated groups to report on healthcare provider vaccine recommendations. While we cannot make direct inferences regarding the positive effects of healthcare provider recommendations with our current data, this finding is supported by guidance in recent HSE programmes, such as the Structured Chronic Disease management programme. As part of this programme, patients undergo a preventative care review where vaccination status is checked and discussed with patients.^49^ A recent study on this programme has found that pneumococcal vaccine recommendations and uptake were higher for enrolled patients (59%) compared with those availing of private care (15%).^50^ While this is a promising finding, making such a programme only available to those with a medical card or GP visit card may only serve to further exacerbate the dual disadvantage faced by those without these healthcare entitlements.

## Conclusions

This study has highlighted the influence that inequities in healthcare entitlements have on an individual’s likelihood to receive a pneumococcal vaccination, and a lack of discussion around pneumococcal vaccination by healthcare professionals with patients. Older adults in Ireland who are entitled to medical care for free at point-of-service are more likely to be vaccinated than those who are faced with the burden of upfront medical costs. Additionally, there is a concerning lack of discussion by healthcare professionals with their patients about the benefits of pneumococcal vaccination. This can lead to a double disadvantage where those who see their primary care providers less frequently are cumulatively less likely to receive vaccine recommendations. To increase vaccination awareness and uptake, healthcare professionals should proactively engage in conversations about pneumococcal vaccination with older adults. This approach would be particularly beneficial in enhancing vaccination rates among those most vulnerable to pneumococcal pneumonia, such as frail individuals and those with at-risk medical conditions.

## Supplementary material

10.1136/bmjph-2025-003996 online supplemental table 1

10.1136/bmjph-2025-003996 online supplemental table 2

10.1136/bmjph-2025-003996 online supplemental table 3

## Data Availability

Data are available on reasonable request.
