# Supplementary material for: Factors associated with pneumococcal vaccination uptake in over 50s in Ireland: a cross-sectional study using results from the Irish Longitudinal Study on Ageing (TILDA)
Source: BMJ Public Health. 2026 Mar 31;4(1):e003996. doi: 10.1136/bmjph-2025-003996 (PMC13052609; doi:10.1136/bmjph-2025-003996)
Supplement: online supplemental table 1 [file bmjph-4-1-s001.pdf]

Supplementary Table S1. Sensitivity analysis of pneumococcal vaccination uptake using robust Poisson regression models with and without the Fried frailty phenotype

Variable	Model 1: Robust Poisson regression (IRR) with Fried excluded	Model 2: Robust Poisson regression (IRR) with Fried included
**Age**		
50-64 years	Ref	Ref
65-74 years	1.49 (1.22,1.82)***	1.53 (1.24,1.88)***
>=75 years	1.42 (1.13,1.77)**	1.42 (1.12,1.81)**
**Gender**		
Male	Ref	Ref
Female	1.22 (1.10,1.36)***	1.24 (1.11,1.39)***
**Marital status**		
Married	Ref	Ref
Never married	1.14 (0.94,1.38)	1.09 (0.88,1.34)
Separated/divorced	0.99 (0.77,1.28)	0.97 (0.73,1.30)
Widowed	1.22 (1.08,1.38)**	1.23 (1.08,1.41)**
**Education**		
Primary/none	Ref	Ref
Secondary	1.06 (0.94,1.20)	1.11 (0.97,1.27)
Third/higher	1.02 (0.88,1.18)	1.07 (0.92,1.26)
**Self-rated health**		
Excellent	Ref	Ref
Very good	1.04 (0.84,1.27)	1.01 (0.81,1.25)
Good	1.10 (0.90,1.34)	1.08 (0.86,1.34)
Fair	0.96 (0.77,1.21)	0.92 (0.70,1.19)
Poor	0.98 (0.71,1.35)	1.08 (0.75,1.55)
**At-risk medical status**		
Not at risk	Ref	Ref
At risk	1.48 (1.33,1.65)***	1.41 (1.26,1.58)***
**Influenza vaccination history**		
Not vaccinated for influenza	Ref	Ref
Vaccinated for influenza	8.42 (6.40,11.07)***	9.08 (6.69,12.32)***
**Health coverage**		
No cover	Ref	Ref
Insurance only	0.97 (0.68,1.40)	0.97 (0.64,1.46)
Medical card only	1.61 (1.12,2.31)**	1.65 (1.08,2.51)*
Dual cover	1.89 (1.32,2.71)***	1.87 (1.24,2.83)**
**GP distance in quantiles**		
1 (Closest proximity)	Ref	Ref
2	0.93 (0.80,1.09)	0.94 (0.79,1.11)
3	0.82 (0.70,0.96)*	0.83 (0.70,1.00)*
4	0.88 (0.75,1.03)	0.93 (0.79,1.10)
5 (Furthest proximity)	0.85 (0.72,1.00)	0.85 (0.71,1.01)
**Fried Frailty phenotype**		
Non-frail	Ref	Ref
Pre-frail	-	1.11 (0.98,1.26)
Frail	-	1.10 (0.89,1.35)
**Model diagnostics**		
Observations (*N*)	4,816	4,026
Wald *F* statistic	(21, 587) = 33.04	(23, 574) = 26.62

Variable	Model 1: Robust Poisson regression (IRR) with Fried excluded	Model 2: Robust Poisson regression (IRR) with Fried included
Prob > *F* (*p* value)	0.00	0.00

IRR, incidence rate ratio; CI, confidence interval; * $p < 0.05$, ** $p < 0.01$, *** $p < 0.001$
