# Supplementary material for: Factors associated with pneumococcal vaccination uptake in over 50s in Ireland: a cross-sectional study using results from the Irish Longitudinal Study on Ageing (TILDA)
Source: BMJ Public Health. 2026 Mar 31;4(1):e003996. doi: 10.1136/bmjph-2025-003996 (PMC13052609; doi:10.1136/bmjph-2025-003996)
Supplement: online supplemental table 2 [file bmjph-4-1-s002.pdf]

Supplementary Table S2. Sensitivity analysis of pneumococcal vaccination recommendation using robust Poisson regression models with and without the Fried frailty phenotype

Variable	Model 1: Robust Poisson regression (IRR) with Fried excluded	Model 2: Robust Poisson regression (IRR) with Fried included
**Age**		
50-64 years	Ref	Ref
65-74 years	1.69 (1.26,2.26)***	1.71 (1.24,2.36)**
>=75 years	1.13 (0.75,1.71)	1.22 (0.78,1.92)
**Gender**		
Male	Ref	Ref
Female	1.73 (1.36,2.19)***	1.72 (1.35,2.21)***
**Marital status**		
Married	Ref	Ref
Never married	1.06 (0.71,1.59)	1.19 (0.77,1.84)
Separated/divorced	0.81 (0.52,1.27)	0.82 (0.49,1.37)
Widowed	1.18 (0.87,1.59)	1.26 (0.91,1.73)
**Education**		
Primary/none	Ref	Ref
Secondary	1.18 (0.87,1.59)	1.12 (0.81,1.56)
Third/higher	1.12 (0.81,1.54)	1.09 (0.76,1.55)
**Self-rated health**		
Excellent	Ref	Ref
Very good	0.88 (0.61,1.27)	0.96 (0.64,1.43)
Good	1.05 (0.73,1.49)	1.16 (0.79,1.69)
Fair	0.89 (0.56,1.41)	1.17 (0.70,1.95)
Poor	0.99 (0.48,2.04)	1.17 (0.47,2.95)
**At-risk medical status**		
Not at risk	Ref	Ref
At risk	1.56 (1.22,1.99)***	1.62 (1.24,2.12)***
**Influenza vaccination history**		
Not vaccinated for influenza	Ref	Ref
Vaccinated for influenza	0.99 (0.78,1.24)	1.07 (0.83,1.38)
**Health coverage**		
No cover	Ref	Ref
Insurance only	1.22 (0.77,1.93)	1.33 (0.79,2.24)
Medical card only	1.35 (0.85,2.15)	1.43 (0.85,2.42)
Dual cover	1.63 (0.99,2.67)	1.83 (1.05,3.19)*
**GP distance in quantiles**		
1 (Closest proximity)	Ref	Ref
2	0.84 (0.60,1.16)	0.98 (0.69,1.40)
3	0.75 (0.53,1.05)	0.72 (0.48,1.07)
4	0.79 (0.57,1.09)	0.95 (0.67,1.37)
5 (Furthest proximity)	0.82 (0.60,1.12)	0.95 (0.67,1.35)
**Fried Frailty phenotype**		
Non-frail	Ref	Ref
Pre-frail	-	0.82 (0.64,1.06)
Frail	-	0.53 (0.30,0.94)*
**Model diagnostics**		
Observations (*N*)	3674	3092

Wald *F* statistic	(21, 583) = 4.33	(23, 566) = 3.77
Prob > *F* (*p* value)	<0.001	<0.001

IRR, incidence rate ratio; CI, confidence interval; * $p < 0.05$, ** $p < 0.01$, *** $p < 0.001$
