# Supplementary material for: Factors associated with pneumococcal vaccination uptake in over 50s in Ireland: a cross-sectional study using results from the Irish Longitudinal Study on Ageing (TILDA)
Source: BMJ Public Health. 2026 Mar 31;4(1):e003996. doi: 10.1136/bmjph-2025-003996 (PMC13052609; doi:10.1136/bmjph-2025-003996)
Supplement: online supplemental table 3 [file bmjph-4-1-s003.pdf]

**Supplementary Table S3.** Sensitivity Analysis of Pneumococcal Vaccination Uptake Determinants Using Robust Poisson, Negative Binomial and Logistic Regression Models

| Variable | Model 1: robust Poisson regression (IRR) | Model 2: negative binomial regression (IRR) | Model 3: logistic regression (OR) |
|---|---|---|---|
| **Age** | | | |
| 50-64 years | Ref | Ref | Ref |
| 65-74 years | 1.53 (1.24,1.88)$^{***}$ | 1.53 (1.24,1.88)$^{***}$ | 1.85 (1.38,2.48)$^{***}$ |
| >=75 years | 1.42 (1.12,1.81)$^{**}$ | 1.42 (1.12,1.81)$^{**}$ | 1.66 (1.16,2.39)$^{**}$ |
| **Gender** | | | |
| Male | Ref | Ref | Ref |
| Female | 1.24 (1.11,1.39)$^{***}$ | 1.24 (1.11,1.39)$^{***}$ | 1.46 (1.19,1.78)$^{***}$ |
| **Marital status** | | | |
| Married | Ref | Ref | Ref |
| Never married | 1.09 (0.88,1.34) | 1.09 (0.88,1.34) | 1.15 (0.80,1.66) |
| Separated/divorced | 0.97 (0.73,1.30) | 0.97 (0.73,1.30) | 0.95 (0.60,1.51) |
| Widowed | 1.23 (1.08,1.41)$^{**}$ | 1.23 (1.08,1.41)$^{**}$ | 1.52 (1.16,1.99)$^{**}$ |
| **Education** | | | |
| Primary/none | Ref | Ref | Ref |
| Secondary | 1.11 (0.97,1.27) | 1.11 (0.97,1.27) | 1.20 (0.94,1.53) |
| Third/higher | 1.07 (0.92,1.26) | 1.07 (0.92,1.26) | 1.13 (0.86,1.48) |
| **Self-rated health** | | | |
| Excellent | Ref | Ref | Ref |
| Very good | 1.01 (0.81,1.25) | 1.01 (0.81,1.25) | 1.02 (0.72,1.44) |
| Good | 1.08 (0.86,1.34) | 1.08 (0.86,1.34) | 1.14 (0.80,1.63) |
| Fair | 0.92 (0.70,1.19) | 0.92 (0.70,1.19) | 0.85 (0.55,1.32) |
| Poor | 1.08 (0.75,1.55) | 1.08 (0.75,1.55) | 1.18 (0.59,2.35) |
| **At-risk medical status** | | | |
| Not at risk | Ref | Ref | Ref |
| At risk | 1.41 (1.26,1.58)$^{***}$ | 1.41 (1.26,1.58)$^{***}$ | 1.87 (1.52,2.30)$^{***}$ |
| **Influenza vaccination history** | | | |
| Not vaccinated for influenza | Ref | Ref | Ref |
| Vaccinated for influenza | 9.08 (6.69,12.32)$^{***}$ | 9.08 (6.69,12.32)$^{***}$ | 14.58 (10.52,20.20)$^{***}$ |
| **Health coverage** | | | |
| No cover | Ref | Ref | Ref |
| Insurance only | 0.97 (0.64,1.46) | 0.97 (0.64,1.46) | 0.96 (0.57,1.63) |
| Medical card only | 1.65 (1.08,2.51)$^{*}$ | 1.65 (1.08,2.51)$^{*}$ | 1.99 (1.14,3.47)$^{*}$ |
| Dual cover | 1.87 (1.24,2.83)$^{**}$ | 1.87 (1.24,2.83)$^{**}$ | 2.58 (1.50,4.45)$^{***}$ |
| **GP distance in quantiles** | | | |
| 1 (Closest proximity) | Ref | Ref | Ref |
| 2 | 0.94 (0.79,1.11) | 0.94 (0.79,1.11) | 0.88 (0.64,1.20) |
| 3 | 0.83 (0.70,1.00)$^{*}$ | 0.83 (0.70,1.00)$^{*}$ | 0.72 (0.52,0.99)$^{*}$ |
| 4 | 0.93 (0.79,1.10) | 0.93 (0.79,1.10) | 0.87 (0.64,1.18) |
| 5 (Furthest proximity) | 0.85 (0.71,1.01) | 0.85 (0.71,1.01) | 0.74 (0.54,1.01) |
| **Fried Frailty phenotype** | | | |
| Non-frail | Ref | Ref | Ref |

| Variable | Model 1: robust Poisson regression (IRR) | Model 2: negative binomial regression (IRR) | Model 3: logistic regression (OR) |
|---|---|---|---|
| Pre-frail | 1.11 (0.98,1.26) | 1.11 (0.98,1.26) | 1.19 (0.96,1.49) |
| Frail | 1.10 (0.89,1.35) | 1.10 (0.89,1.35) | 1.19 (0.81,1.77) |
| **Model diagnostics** | | | |
| Observations (*N*) | 4,026 | 4,026 | 4,026 |
| Wald *F* statistic | (23, 574) = 26.62 | (23, 574) = 26.62 | (23, 574) = 21.91 |
| Prob > *F* (*p* value) | <0.001 | <0.001 | <0.001 |
| Alpha ($\alpha$) | - | 3.04e-33 | |

*IRR, incidence rate ratio; CI, confidence interval; OR, Odds ratio;* $^{*}$ $p < 0.05$, $^{**}$ $p < 0.01$, $^{***}$ $p < 0.001$
